# Editorial: The gut and the brain – with focus on autism spectrum disorders

**DOI:** 10.3402/mehd.v23i0.18984

**Published:** 2012-08-24

**Authors:** Tore Midtvedt

That was the title of a one-day well attended symposium held in May 2012 at the Nobel Forum of the Karolinska Institute in Stockholm. Scientists, therapists and parents met and listened to a number of highly interesting lectures describing the multi-faceted picture of ASD and the challenges as well as the encouraging results of ongoing research in the field. The collection of extended abstract pertaining to this symposium is now published *in Microbial Ecology in Health and Disease*.

Throughout the day the autistic child was in focus – first presented in a documentary from Canada, and then followed by a successful never-give-up story from the mother of three autistic children (1). The two following lectures focused on the complexity of phenotypic and genotypic aspects of autism (2, 3), which was further underlined by Heijtz (4) in his lecture describing animal experiments in which both environmental factors and prenatal stress were seen to impact on the development of social and motor functions in off-springs. To study deviations and alterations in autistic children, Hugdahl (5) then showed how he and his group by combining advanced nuclear magnetic imaging with functional spectroscopy could quantify regional concentrations of substances acting as neurotransmitters.

The possibility that it is the gut that triggers the production of neurotransmitters as well as other bioactive compounds was underlined in the lecture by myself (6), which was followed by an exciting lecture by Bienenstock (7) focusing on the communicative side of that possibility, i.e. that *Nervus vagus* may act as a highway from gut to brain. Altered intestinal metabolic functions in autism, exemplified by quantitative differences in urinary excretion from many compounds, was the scope of the lecture by Nicholson (8) who focused on the hypothesis that some sulfur-containing compounds may act as neurotransmitters.

In his lecture, Reichelt (9) showed that dietary derived, bioactive peptides are present in urine from autistic children, most often deriving from intestinal degradation of casein. The role of the gut microflora in mitochondrial dysfunctions was highlighted in an exciting lecture by MacFabe (10), and Christophersen (11), in the final lecture of the day, posed the interesting question: Should autism be considered a canary bird telling that *Homo sapiens* may be on its way to extinction?

In the ensuing plenary discussion the focus was on the mechanism(s) underlying the increased incidence of autism. Two major scenarios were presented. If the increased incidence is real, it might reflect either that the average mutation rate in large populations, such as e.g. the United States, has increased, or that so far unknown environmental factors act on already existing metabolic pathways. Whatever the mechanisms, the urgent need for further research was underlined. On an individual level – and based on the experience of the participants in the panel – an encouraging message was given to the parents of an autistic child: Autism is treatable-symptoms can be reduced and behavior can be changed.

*Tore Midtvedt* Editor-in-Chief
